# Modeling Poliovirus Infection Using Human Engineered Neural Tissue Enriched With Motor Neuron Derived From Embryonic Stem Cells

**DOI:** 10.3389/fcell.2020.593106

**Published:** 2021-01-06

**Authors:** Érika Cosset, Youssef Hibaoui, Sten Ilmjärv, Pierre-Yves Dietrich, Caroline Tapparel, Karl-Heinz Krause

**Affiliations:** ^1^Laboratory of Tumor Immunology, Department of Oncology, Center for Translational Research in Onco-Hematology, Geneva University Hospitals, University of Geneva, Geneva, Switzerland; ^2^Service de Gynécologie et Obstétrique, HFR Fribourg -Hôpital Cantonal, Fribourg, Switzerland; ^3^Department of Microbiology and Molecular Medicine, Medical School, University of Geneva, Geneva, Switzerland

**Keywords:** poliovirus type III, human embryonic stem cells, tissue engineering, motor neuron differentiation, neural differentiation, disease modeling, pluripotent stem cells

## Abstract

Poliomyelitis is caused by poliovirus (PV), a positive strand non-enveloped virus. Since its discovery in the 1950s, several cell culture and molecular methods have been developed to detect and characterize the various strains of PV. Here, we provide an accurate and standardized protocol to differentiate human embryonic stem cells (hESCs) toward engineered neural tissue enriched with motor neurons (MN ENTs). These MN ENTs expressed markers of motor neuron CHAT and Hb-9 as revealed by immunofluorescence staining and quantitative RT-PCR. Interestingly, our results suggest that motor neurons are responsible for the permissiveness of poliovirus within the MN ENTs. Moreover, our study revealed the molecular events occurring upon PV-3 infection in the MN ENTs and highlighted the modulation of a set of genes involved in EGR-EP300 complex. Collectively, we report the development of a reliable *in vitro* model to investigate the pathophysiology of PV infection, allowing to both design and assess novel therapeutic approaches against PV infection.

## Introduction

Poliovirus (PV) is a small non-enveloped virus with a single-strand positive genomic RNA classified in the *Enterovirus C* species of the *Picornaviridae* family (Couderc et al., 1989). Due to its fecal-oral transmission route, PV infection is most often present in high density population with subpar sanitation systems (Falconer and Bollenbach, 2000). PV specifically infects the gray matter of the anterior horn of the spinal cord, from which its name derived (Greek polios = gray, myelos = matter). Indeed, in 1–2% of infected individuals, the virus enters the central nervous system (CNS) and replicates in motor neurons (MN) within the spinal cord, brain stem, or motor cortex (Kew et al., 2005; Racaniello, 2006). The motor function impairment leads to muscle paralysis, also known as acute flaccid paralysis, and even death. Consequently, survivors of poliomyelitis often remain disabled (Mueller et al., 2005). Since the 1950's, major vaccination campaigns allowed to decrease the incidence of poliomyelitis (Cochi et al., 2016). Despite few reported cases of poliomyelitis, several reports acknowledge the possible eradication of PV in the next decade (Kennedy et al., 2015). To date, no efficient antiviral treatments are available for PV infection despite significant efforts (Arita et al., 2011; van der Sanden et al., 2016). Frequently explored antiviral targets are the viral capsid proteins and non-structural proteins (2A, 2C, 3C, and 3D). Host-targeting antiviral strategies are also being investigated, such as those targeting factors necessary for PV replication, namely eIF4A, GBF1, and VCP/p97 (Molla et al., 1993; Shimizu et al., 2000; de Palma et al., 2008; Arita et al., 2012).

Even though neurological symptoms are rare complications of the PV infection, the molecular mechanisms underlying poliomyelitis occurrence are poorly understood. This is remarkable considering that PV represents one of most thoroughly studied and best-understood virus models. The mouse models have been widely used to gain insights into PV infection processes however, genetic, immune, and physiological differences between humans and mice limit their value (Jubelt et al., 1980). Moreover, mice are only susceptible to certain adapted strains of PV, and transgenic mice expressing the PV receptor CD155 cannot be infected orally and thus require infection via nasal or intramuscular routes to induce paralytic disease (Crotty et al., 2002). Alternatively, non-human primates such as Bonnet Monkeys have been extensively used to study PV pathogenesis, as infection of these monkeys results in a limb paralysis that mimics in some extent human paralytic poliomyelitis both clinically and pathologically (John et al., 1992). Unfortunately, these models cannot accurately recapitulate the specificities of PV infection in the human brain.

The discovery that human pluripotent stem cells (PSCs) can be differentiated into 3D culture systems including engineered neural tissues (ENTs) and organoids has provided unprecedented opportunities to investigate diseases affecting the CNS (Hibaoui and Feki, 2012; Lancaster et al., 2013; Cosset et al., 2015). Several studies have been successful in generating ENTs and organoids from disease-specific induced pluripotent stem cells (iPSCs) or human embryonic stem cells (hESCs), providing excellent *in vitro* models for pathophysiology and drug screening (Hibaoui and Feki, 2012; Cosset et al., 2015; Giandomenico et al., 2019). In this context, ENTs and organoids derived from human PSCs have also proven to be reliable models for studying infectious diseases (Cosset et al., 2015; D'Aiuto et al., 2019), including the use of cerebral organoids to model microcephaly caused by Zika virus infection (Cugola et al., 2016; Garcez et al., 2016; Qian et al., 2016).

We report herein the development of an *in vitro* model of human ENT enriched with motor neurons from hESCs. Using this model, we sought to better understand the mechanisms underlying PV-3 tropism and the molecular events following viral infection in this *in vitro* model.

## Materials and Methods

### Maintenance of hESCs

hESC cell lines H1 and H9 (supplied by WiCell Research Institute) were cultured in feeder-free conditions as described previously (Cosset et al., 2015, 2016), and maintained in a humidified 37°C, 5% CO2 incubator. These hESCs were plated onto pre-coated Matrigel (CELLstartTM, Invitrogen) and cultured in Nutristem medium (Stemcell Biotechnologies) with media changes every 2 days.

### Engineered Neural Tissue Enriched With Motor Neurons (MN ENTs)

ENTs were generated as previously described (Cosset et al., 2015, 2016), with some modifications to direct toward a motor neuron fate. hESC were dissociated with accutase (Gibco, Thermo Fisher Scientific), resuspended in medium containing 2 μM ROCK inhibitor (Y-27632) (Abcam Biochemicals) to improve cell survival, and allowed to aggregate in Aggrewell™ dishes for 24 h. Then, these aggregates were transferred into low attachment wells (Costar, Corning Life Sciences) in N2B27 neural medium consisting in equal parts of DMEM-F12 and Neurobasal, 0.5% N2, 1% B27, and 2 mM L-glutamine, 10 μM β-mercaptoethanol, 1% non-essential amino acids, 50 U/ml penicillin, and 50 μg/ml streptomycin (all from Gibco, Thermo Fisher Scientific). As outlined in Supplementary Figure 1B, and from the first day to day 5, N2B27 medium was supplemented with the Dual-Smad cocktail: SB431542 (10 nM) and LDN (4 nM) (from Cell Guidance). From day 5 to day 12, N2B27 medium was supplemented with 1 μM retinoic acid (RA). From day 12 to day 18, N2B27 medium was supplemented with 1 μM RA and 100 ng/mL sonic hedgehog (shh). From day 18 to day 26, N2B27 medium was supplemented with 10 ng/mL glial cell-derived neurotrophic factor (GDNF), 10 ng/mL brain-derived neurotrophic factor (BDNF), 10 ng/mL insulin growth factor (IGF-1), and 100 ng/mL shh (all from R&D Systems, Inc.,). At day 26, N2B27 medium was supplemented with 1 μM CpE, a γ-secretase inhibitor, (from Cell Guidance) for 1 day. At day 27, these aggregates were plated on a hydrophilic polytetrafluoroethylene (PTFE) membrane (6 mm diameter, 0.4 lm; BioCellInterface) for 2 weeks in N2B27 medium with media changes every 2 days.

### Non-directed Late Neural Engineered Neural Tissues (lnENTs)

Neural ENTs were generated as previously described (Cosset et al., 2016), with some minor modifications. hESC were dissociated with accutase (Gibco, Thermo Fisher Scientific), resuspended in medium containing 2 μM ROCK inhibitor (Y-27632) (Abcam Biochemicals) to improve cell survival, and allowed to aggregate in Aggrewell™ dishes for 24 h. Then, these aggregates were transferred into low attachment wells (Costar, Corning Life Sciences) in N2B27 neural medium consisting in equal parts of DMEM-F12 and Neurobasal, 0.5% N2, 1% B27, and 2 mM L-glutamine, 10 μM β-mercaptoethanol, 1% non-essential amino acids, 50 U/ml penicillin, and 50 μg/ml streptomycin (all from Gibco, Thermo Fisher Scientific). As outlined in Supplementary Figure 1C, from the first day to day 5, N2B27 medium was supplemented with the dual-Smad cocktail: SB431542 (10 nM) and LDN (4 nM) (from Cell Guidance). From day 5 to day 12, N2B27 medium was supplemented with 10 ng/mL EGF and 10 ng/mL FGF (all from R&D Systems, Inc.,). From day 12 to day 19, N2B27 medium was supplemented 10 ng/mL GDNF and 10 ng/mL BDNF (all from R&D Systems, Inc.,). From day 19 to day 26, N2B27 medium was supplemented with 10 ng/mL GDNF, 10 ng/mL BDNF, and 1 μM CpE (from Cell Guidance). At day 26, these aggregates were plated on a hydrophilic polytetrafluoroethylene (PTFE) membrane (6 mm diameter, 0.4 lm; BioCellInterface) for 2 weeks in N2B27 medium with media changes every 2 days.

### Non-directed Early Neural Engineered Neural Tissues (enENTs)

Immature ENT, named enENT, were generated as previously described (Cosset et al., 2015).

### Viruses

PV-3 (Sabin strain) was propagated on Vero cells in a 5% CO_2_-containing atmosphere in DMEM (Gibco) supplemented with 2 mM L-glutamine, 100 μg/ml penicillin-streptomycin, 1 μg/ml amphotericin B, 100 μg/ml gentamicin, 10% FCS, 0.2% NaHCO_3_ and 2% Hepes. EV-71 was propagated as previously described (Tseligka et al., 2018). PV-3 was inoculated on cells for 1 h at 37°C, then the inoculum was removed. Cells were washed once with PBS and left at 37°C until the appearance of a strong cytopathic effect. Viral supernatant was collected, clarified by centrifugation (5′, 1,500 RPM at 4°C), aliquoted and frozen at −80°C. The cell culture infective dose 50 (CCID50)/mL was determined for each viral stock using the Karber method (G, 1931). Infection was performed at a MOI of 1 by addition of diluted viral supernatant directly into the medium for monolayer cultures, or on the top of the ENT for three dimensional cultures over 2 h. Medium exchange was performed every 2 days.

### Immunofluorescence and Immunochemistry

Immunofluorescence and immunochemistry were performed as previously described (Cosset et al., 2015, 2016). The following primary antibodies were used: mouse anti-neuronal nuclei-specific protein (NeuN) (Chemicon;MAB377), rabbit anti-βIII-tubulin (Covance;PRB435P), goat anti-ChaT (Chemicon; AB144P), rabbit anti-HB-9 (Abcam; ab922606), rabbit anti-ISLET1 (Abcam; ab22450), goat anti-GALR3 (Abcam), mouse anti-EV-71 (Abcam; ab36367), and mouse anti-PV-3 (Abcam; ab22450). Alexa Fluor (555 and 488)-labeled antibodies from goat or donkey against mouse, goat, or rabbit (Molecular Probes) were used as secondary antibodies. Cell nuclei were stained with DAPI (4, 6-diamidino-2-phenylindole). For IHC, biotin-conjugated anti-rabbit IgG or anti-goat IgG were used and developed using avidin-biotin peroxidase detection system (Vector Labs) with 3,3′-diaminobenzidine substrate (DAB, Sigma-Aldrich) after 2 min of incubation.

### Quantitative Real-Time PCR

RNA extraction and cDNA synthesis were, respectively, performed using the Rneasy Mini Kit (Qiagen) and Takara Kit for cDNA synthesis primed with Oligo(dt) and Random Primers according to manufacturer's instructions. SYBRGreen reagent was used for Real-time PCR on the ABI Prism 7000 sequence detection system (Applied Biosystems) according to the manufacturer's instructions. ALAS1 and EEF1 were used as housekeeping genes. The results were analyzed using the 2-ΔΔCt method. Primer sequences are provided in the Supplementary Table 1. All experiments were performed, at least, in triplicate.

### Real-Time (RT)-PCR Screening for the Presence of PV-3

PV-3 infected and non-infected ENT were screened for the presence of PV-3 with the Entero/Ge/08 real-time PCR (Racaniello, 2006). After tissue homogenization, 400 ul of supernatant were collected for RNA extraction with easyMAG (bioMérieux). Taqman Universal Mastermix (Applied Biosystems) was used to perform the Real-time PCR screening using the StepOne thermocycler (Applied Biosystems).

### PCR Screening for the Presence of PSC, NSC, and MN Markers

For non-quantitative PCR, reactions were performed in a Biometra thermocycler (Göttingen, Germany), with RedTaq polymerase mix (Sigma-Aldrich, St. Louis, MO, USA), 250 nM primers and 2 μL of cDNA. The primer sequences used for non-quantitative RT-PCR are listed in Supplementary Table 1.

### Microarray

The Illumina TotalPrep RNA amplification kit was used to synthetize the first and second strand cDNA, as well as cDNA and cRNA purification according to the manufacturer's instructions. The microarray was performed on human HT-12 v3.0 Illumina microarrays. Preprocessing consisted of a background correction, a log2 transformation and quantile normalization, as previously described (Cosset et al., 2015). Then, the limma package from Bioconductor was used to identify the differentially expressed genes (Gentleman et al., 2004). Finally, g:profiler was used for enriched functional annotation analysis (Raudvere et al., 2019).

### Statistical Analysis

All quantitative data presented are the mean ± SEM. Samples used and the respective *n* values refer to the number of independent experiments and are listed in the figure legends. Statistical analyses were performed with the students *t*-test and ANOVA where *p* < 0.05 was considered significant.

## Results

### Generation and Characterization of Engineered Neural Tissue Enriched With Motor Neuron (MN ENTs)

**Figure 1:**
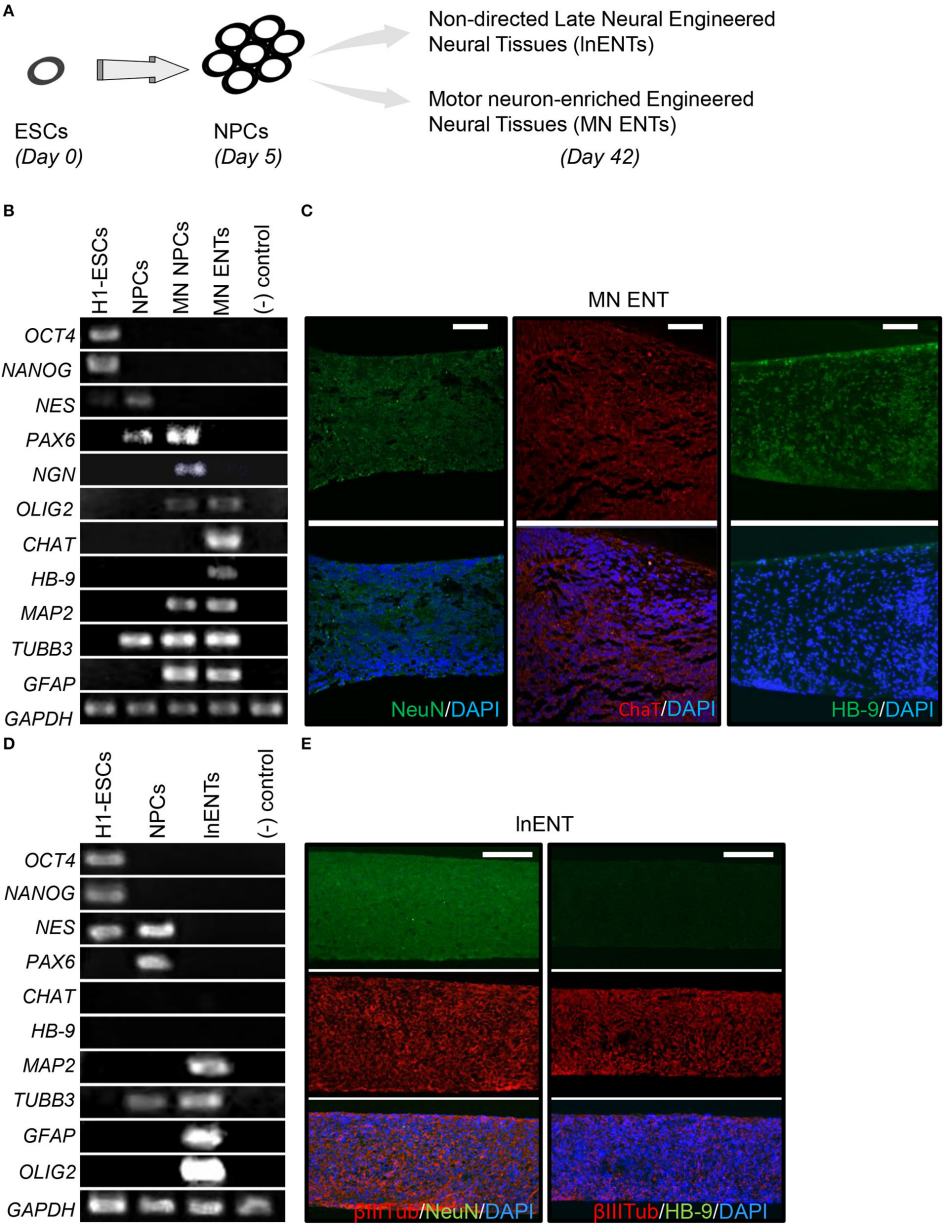
MN ENT generation and characterization. **(A)** Schematic representation of hESC differentiation toward lnENTs and MN ENT. **(B)** RT-PCR analysis of the expression of pluripotency (*OCT4* and *NANOG*), neuroprogenitor cells NPCs (*NES* and *PAX6*), motor neuron progenitor cells (MN NPCs) (*NGN, PAX6*, and *OLIG2*), motor neuron enriched ENTs (*CHAT* and *HB9*), and markers for neuronal, astroglial and oligodendroglial cells (*MAP2, TUBB3, GFAP*, and *OLIG2*). Human lymphocytes B were used as negative controls. **(C)** Immunofluorescence showed NeuN, CHAT, and HB-9-immunoreactive cells present in the whole MN ENT. Scale bar = 50 μm. **(D)** RT-PCR analysis of the expression of pluripotency (*OCT4* and *NANOG*), neuroprogenitor cells NPCs (*NES* and *PAX6*), and non-directed late neural ENTs (*MAP2, TUBB3, GFAP*, and *OLIG2*). Human lymphocytes B were used as negative controls. **(E)** Immunofluorescence showed βIII-Tubulin, NeuN, and HB-9-immunoreactive cells present, or not, in the lnENT. Scale bar = 100 μm.

Given the known tropism of poliovirus for MNs, we developed a protocol to enrich engineered neural tissues with motor neurons (MN ENTs), as outlined in Supplementary Figure 1A. To initiate MN differentiation, colonies of undifferentiated hESCs, expressing *OCT4*, and *NANOG* (Figures 1A,B), were dissociated into single cells and re-aggregated in multiwell plates for 24 h to allow size-calibrated neurospheres (Supplementary Figures 1A–E). As previously described (Chambers et al., 2009; Kriks et al., 2011), dual-smad inhibition was used to improve neural induction during the initial days of culture (Supplementary Figure 1A) (Chambers et al., 2009; Kriks et al., 2011). As illustrated in Supplementary Figures 1A,B, these spheres were induced to differentiate into neuroprogenitor cells (NPCs) in suspension culture (Supplementary Figure 1C). Thereafter, the addition of retinoic acid (RA), known to induce caudalization after the initial neuralization, quickly oriented neural cells toward MN neural progenitors (MN NPCs), expressing *PAX6, NGN*, and *OLIG2* (Figure 1B, Supplementary Figures 1A,C,D). In response to shh, MN sphere NPCs further differentiated into MN expressing HB-9 and CHAT (Figure 1B). Then, GDNF, BDNF, IGF-1 and shh promoted the final differentiation. To improve neural maturation, the γ-secretase inhibitor compound E was added during the final step of the neural maturation phase (Supplementary Figure 1A). Thereafter, these MN NPCs were placed on a semipermeable PTFE membrane to facilitate the final maturation of MN engineered neural tissue (MN ENT) on an air–liquid interface over 2 weeks (Supplementary Figure 1E). To better characterize the model, MN ENTs were analyzed, stained and compared to non-directed late neural ENT (lnENT, enriched for differentiated neurons and astrocytes), for MN and different neuronal markers (Figures 1A–C) (Supplementary Figure 1F). As expected in MN ENTs, we observed an induction of *HB-9* and *CHAT* mRNA detected by RT-PCR (Figure 1B). These MN markers were also expressed in MN ENTs at the protein level as shown by the immunofluorescence analysis (Figure 1C). Such induction of *HB-9* and *CHAT* genes was not observed in lnENTs (Figure 1D). Similarly, lnENTs did not show any expression of HB-9 and CHAT proteins (Supplementary Figure 1F). Moreover, as lnENTs, MN ENTs expressed markers for astroglial and oligodendroglial cells (*GFAP* and *OLIG2*) and general markers for neurons such as *TUBB3, MAP2* as shown by RT-PCR (Figures 1B,D,E). Collectively, our results suggest an efficient enrichment of MN in MN ENTs.

### Motor Neurons Promote the Spread of Poliovirus Infection in MN ENTs

**Figure 2:**
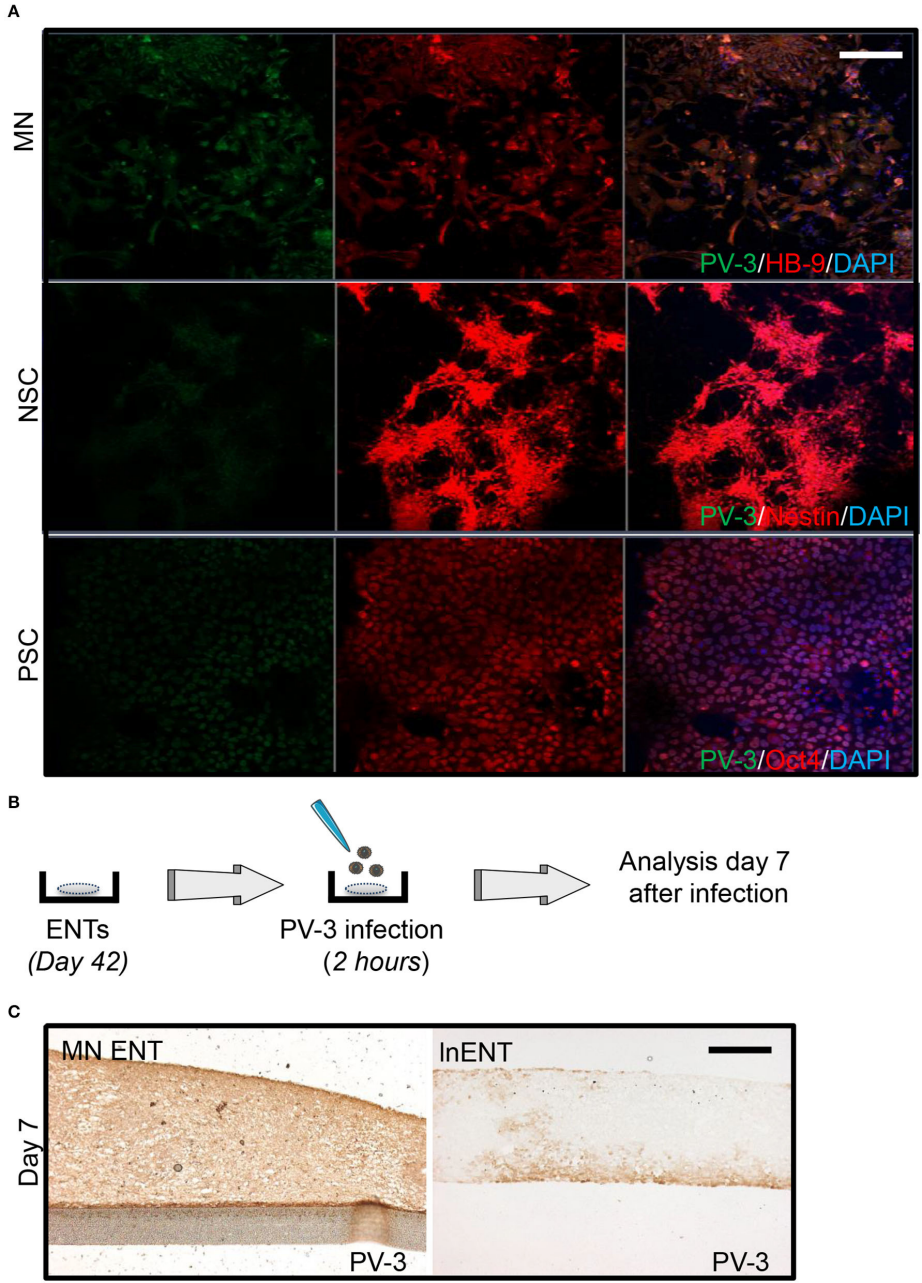
MN ENT shows a greater permissiveness for PV-3 compare to lnENT. **(A)** Infection of motor neurons (MN), neural progenitors (NPC), and human embryonic stem cells (hESCs) by PV-3. Immunofluorescence showed PV-3 (green), HB-9, Nestin, and Oct4 (all in red) in MN, NPC, and PSC, respectively. Scale bar = 50 μm. **(B)** Schematic representation of PV-3 and EV-71 infection of ENTs. **(C)** Immunohistochemistry showing PV-3-immunoreactive cells within MN ENTs (left panel) and lnENT (right panel).

The permissiveness of cells is defined by their potential to support the viral life cycle. Therefore, we sought to investigate PV-3 permissiveness in 2D and 3D culture. We first evaluated PV-3 infection at different stages of hESC differentiation toward MN namely: (i) at the undifferentiated state, (ii) at the NPC level and (iii) in MN. We thus infected all cells by adding PV-3 at a multiplicity of infection (MOI) of 1 over 1 h, then replaced the medium and maintained the cells in culture for 24 h. Our results showed that MNs were highly permissive to PV-3 infection, as evidence by the high proportion of HB-9 positive cells co-stained for PV-3 (Figure 2A). hESCs were less permissive to PV-3 infection, whereas NPCs had none or very few PV-3 immunoreactive cells (Figure 2A). We then compared the permissiveness and spread of PV-3 infection in MN ENTs and in two distinct neural ENT models at day 7 after infection. To do so, we generated both non-directed early neuronal ENTs (enENTs) that resemble the early developing brain and exhibit radially organized cells positive for nestin, musashi, and Pax6; (Cosset et al., 2015) and lnENTs (Figure 1E) (Cosset et al., 2016). On day 7 after infection, we noticed a striking dissemination of PV-3 virus in MN ENTs, whereas lnENTs and enENTs were only weakly infected (Figures 2B,C) (Supplementary Figure 2A). Therefore, differentiation of ENTs into a motor neuron phenotype substantially enhances permissiveness for PV-3 infection. Next, we performed the same infections using enterovirus-71 (EV-71), a neurotropic EV from the A species, known to more generally infect neurons and glial cells (Tseligka et al., 2018). Infection with this virus showed a much more pronounced tropism for lnENTs, while it infected the MN ENTs rather weakly (Supplementary Figure 2B). Similarly, the enENT showed a relatively weak pattern of EV-71 infection (Supplementary Figure 2A). The enENTs were even less permissive to PV-3, with only a few infected cells (Supplementary Figure 2A). Altogether, these results demonstrate an evident permissiveness of MN ENTs for PV-3 infection due to the presence of MN within the tissue.

### Features of Poliovirus Infection in MN ENTs

**Figure 3:**
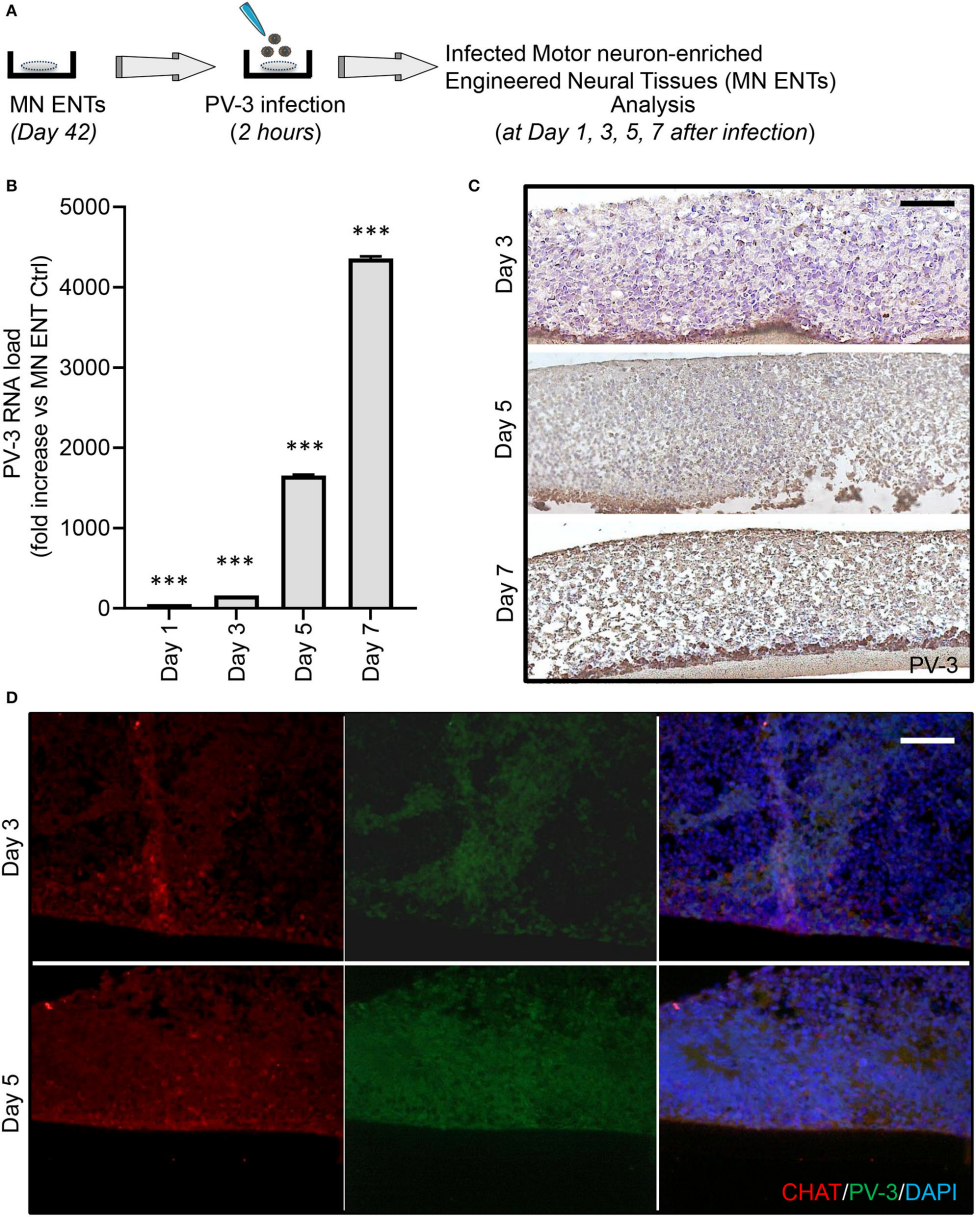
PV-3 preferentially infects motor neurons in MN ENTs. **(A)** Schematic representation of PV-3 infection of MN ENTs. **(B)** Quantitative RT-PCR of mRNA of PV-3 in PV-3-infected MN ENTs at day 1, 3, 5, and 7. Data are normalized to housekeeping genes; mean ± SEM with ***p < 0.01 for *n* = 3 independent experiments. **(C)** MN ENTs were fixed at 3, 5, and 7 days after PV-3 infection, sectioned, stained by immunohistochemistry with antibodies against PV-3. Scale bar = 50 μm. **(D)** MN ENTs were fixed at 3, and 5 days after PV-3 infection, sectioned, and stained with antobodies against PV-3 (in green), CHAT (in red) and with DAPI (in blue). Scale bar = 50 μm.

We first evaluated the kinetics of viral proliferation within MN ENTs at day 1, 3, 5, and 7 after PV-3 infection by quantitative RT-PCR (Figure 3A). As shown in Figure 3B, PV-3 expression increased by 100-, 160-, 1,650-, and 4,200-fold at day 1, 3, 5, and 7 after infection, respectively. In line with this, the immunohistological analysis of PV-3 infected MN ENTs revealed that PV-3 was able to penetrate deep into the tissue, suggesting that tissue organization and extracellular matrix do not impede viral infection and dissemination (Figure 3C). A time course of PV-3 infection revealed viral multiplication, at days 2–3 and presence in the entire tissue by days 5 and 7 after infection, as detected by both immunochemistry and quantitative RT-PCR, respectively (Figures 3B,C). We also observed a tissue damage starting from day 5 to 7 after infection (Figure 3C). Of note, immunofluorescence analysis revealed the co-staining of PV-3 with markers of MN within MN ENTs including CHAT, HB-9, and ISLET1, supporting the notion that MNs within MN ENTs are responsible for the permissiveness to PV-3 infection (Figure 3D) (Supplementary Figure 3A).

### Differentiated Neurons Are More Permissive and Sensitive to PV-3 Infection

**Figure 4:**
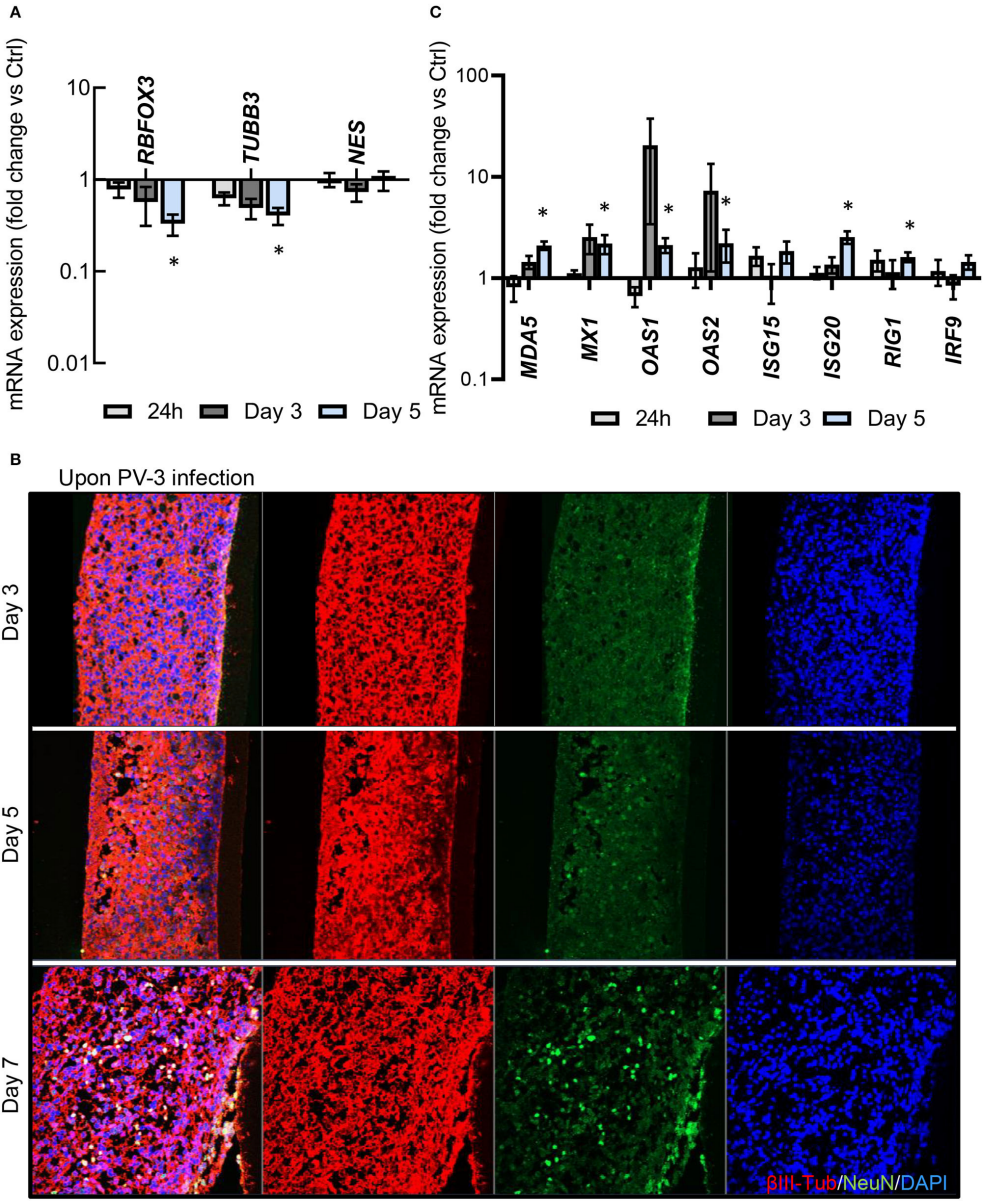
PV-3 infection also affects neural progenitors and induces an innate antiviral response. **(A)** *RBFOX3, NES, and TUBB3* mRNA expression was determined by qPCR in PV-3-infected MN ENT at day 1, 3, and 5. **(B)** Immunofluorescence showing βIII-Tubulin, and NeuN -immunoreactive cells within the MN ENTs at day 3, 5, and 7. **(C)** Quantitative RT-PCR analysis of interferon-related or interferon stimulated mRNA expression in by qPCR in PV-3-infected MN ENTs at day 1, 3, and 5. Data are represented as mean (*n* = 3) ± SEM (*p < 0.05).

We next investigated the kinetics of RNA levels of selected markers for neural progenitors (*NES* for nestin) and for markers of mature neurons (*TUBB3* and *RBFOX3* for β3-tubulin and NeuN, respectively) at 24 h, day 3 and 5 after infection, detected by quantitative RT-PCR (Figure 4A). Our results revealed a time-dependent decrease of *RBFOX3* and *TUBB3* expression following PV-3 infection, while *NES* expression remained unchanged (Figures 4A,B) (Supplementary Figure 4A). The results thus indicate that mature neurons are more sensitive to PV-3 infection than neural progenitors. Given that interferon (IFN) signaling has been shown to control tissue tropism and pathogenicity of PV (Ida-Hosonuma et al., 2005), we further investigated the kinetics of RNA levels of IFN-related and -stimulated genes, in response to PV-3 infection by quantitative RT-PCR (Figure 4C). Interestingly, *MDA-5* (for melanoma differentiation-associated gene 5), *MX1* (for myxovirus resistance 1), *OAS1* (for oligoadenylate synthetase 1), *ISG20* (for Interferon Stimulated Exonuclease Gene 20) and *RIG-1* (for reticnoic acide-inducible gene 1) were up-regulated at day 5 after infection (Figure 4C). This is a notable finding since these genes are recognized as being interferon-inducible antiviral effectors (Sadler and Williams, 2008). Collectively, these results indicated that the MN ENT model recapitulates the innate immune response and probably the first line of defense against viral infection. Surprinsingly though, this response appears rather late, at a moment where a substantial number of mature neurons are already damaged (Figures 3B, 4B; bottom panel).

### Transcriptomic Analysis of PV-3 Infected MN ENTs

**Figure 5:**
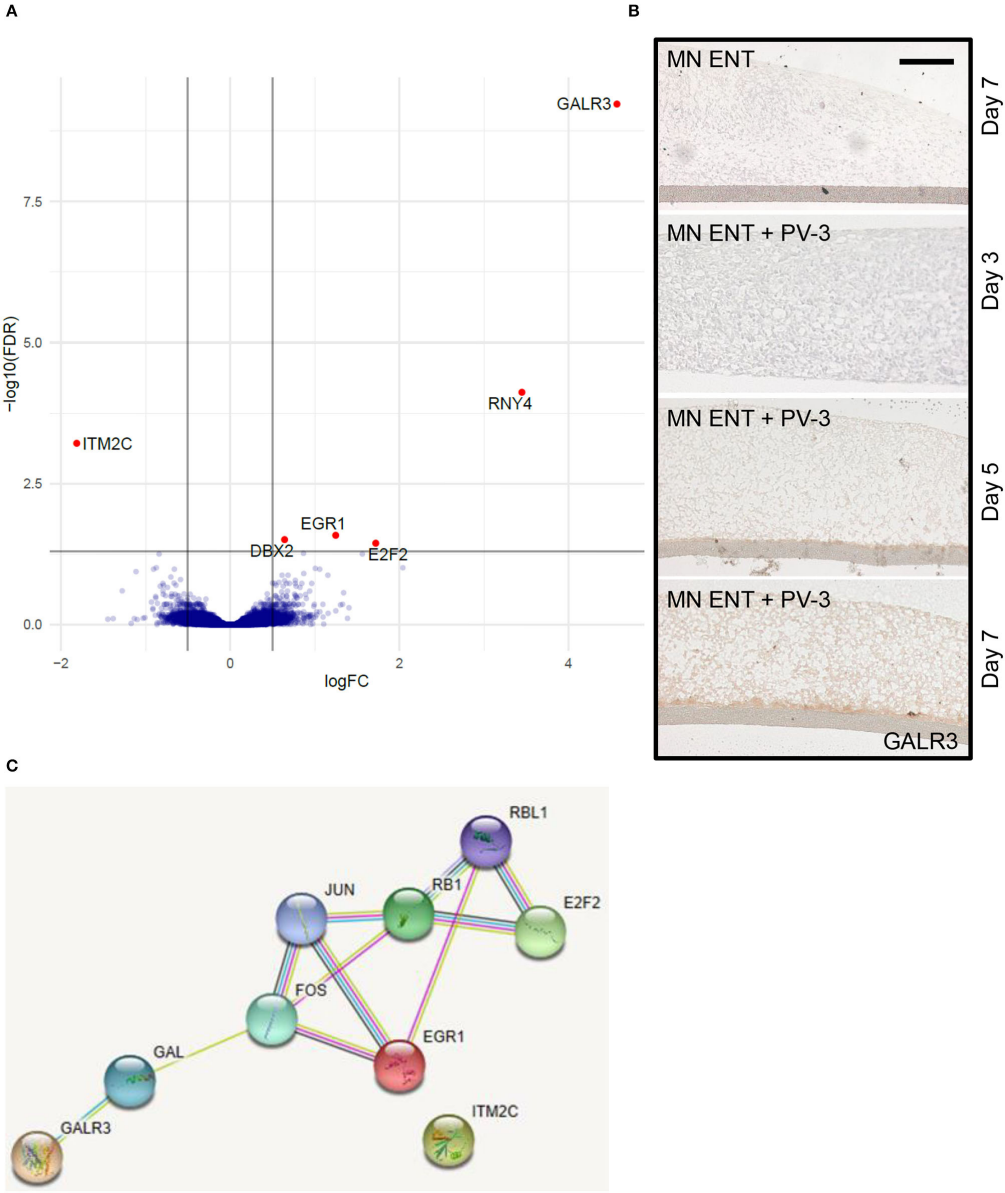
PV-3 infection induces the expression of genes involved in EGR-EP300 complex. **(A)** Volcano plot representing the differentially expressed genes upon PV-3 infection at day 5. **(B)** Immunohistochemistry showed GALR3-immunoreactive cells present in uninfected MN ENT (top panel) and infected MN ENT at day 3, 5, and 7. **(C)** String analysis shows known and predicted interactions between identified genes expressed upon PV-3 infection.

To get more insight into the molecular mechanisms following PV-3 infection in MN ENTs, we compared the transcriptomic gene expression profiles of MN ENTs infected with PV-3 to non-infected controls. Microarray analysis revealed a set of six differentially expressed genes with five upregulated genes (*GALR3*, for galanin receptor type 3; *RNY4*, for RNA Ro-associated Y4; *EGR1*, for early growth response 1; *DBX2*, for developing brain homeobox 2; *E2F2*, for E2F transcription factor 2) and one downregulated gene (*ITM2C*, for integral membrane protein 2C) (Figure 5A) (Supplementary Table 2). Immunostaining of MN ENTs confirmed the increased expression of GALR3 in infected MN ENTs over time (Figure 5B). Gene ontology enrichment analysis (https://biit.cs.ut.ee/gprofiler/gost) provided evidence that suggests expression of genes involved in the EGR-EP300 complex. STRING analysis (https://string-db.org/), presenting known and predicted protein-protein direct or indirect interactions, showed interactions between E2F2, EGR1, and GALR3 (Figure 5C). However, gene expression changes through PV were highly selective for a few genes, and consequently multidimensional scaling analysis confirmed a high similarity between PV-3 infected and control MN ENT (Supplementary Figure 4B).

## Discussion

The discovery that human embryonic and induced pluripotent stem cells can be differentiated into engineered neural tissues has provided unique opportunities to study diseases and infections affecting the CNS. In our previous study, by using *in vitro* ENTs and biopsy samples from infected fetal brains, we could establish that human cytomegalovirus (HCMV) shows a high tropism for early migrating neurons, providing strong evidence that ENT is a suitable physiological model for studying viral infection. Here, we similarly show that human ENTs enriched with motor neuron can be used as a reliable and relevant model for studying PV infection. We demonstrate that motor neurons are required for a high level ENT infection by PV. Following infection, a decrease in mature motor neurons was observed, suggesting motor neuron cytotoxicity due to PV infection. Transcriptomic analysis revealed highly significant changes in expression of a limited number of genes, rather than a massive gene dysregulation.

A striking finding from our study is the extent of viral infection and virion release. Immunohistochemistry showed a large majority of cells within MN ENTs to be positive for viral antigens. This is in contrast to one of our previous reports using HCMV, where only a small minority of cell was infected (Cosset et al., 2015). Similarly, quantitative RT-PCR of virion release revealed large amounts of viral RNA, far above values that we observed previously with CMV. Thus, not only is our tissue model highly permissive to PV infection, it also provides a means to investigate the aggressive nature of viral dissemination in the appropriate tissue context.

Surprisingly, despite the widespread infection with the virus, changes in the transcriptome remained limited to only a few genes: *GALR3, EGR1, E2F2, RNY4, DBX2* were found upregulated, and *ITM2C* was downregulated. Among those, *GALR3*, encodes a G-protein-coupled receptor with widespread distribution in the brain. It plays a role in several physiologic processes (such as sensory/pain processing, hormone secretion, cognition/memory, and feeding behavior) (Lang et al., 2007). There are indications that GALR3 might be involved in CNS infection, since a selective increase in galanin-positive neurons has been shown in a mouse model of sensory ganglia infection with herpes simplex virus (HSV1 and HSV2) (Henken and Martin, 1992a,b). It remains to be seen whether this upregulation of GALR3 expression is part of the host response or due to manipulation of the host tissue by viruses.

*E2F2* and *EGR1* are both involved in the EGR-EP300 complex. The latter encodes for a transcription factor (EGR1), which will form a complex with EP300 and ultimately lead to expression of various target genes, including *E2F2*. Notably, EP300 regulates transcription via chromatin remodeling and is important in cell proliferation and differentiation processes. EP300 is a target of several viruses, including PV, which has been shown to affect the CREB-dependent pathway (Yalamanchili et al., 1997). EP300 knock-down has also been suggested to improve production of PV vaccine (van der Sanden et al., 2016).

In this study, we also documented upregulated genes associated with cellular host responses to viral infection, more specifically the type I interferons, IFNα/β (Desmyter et al., 1968). Of note, previous studies have reported that PV interferes with IFN responses by cleaving the host MDA5 and MAVS proteins, two RIG-I-like receptors (Feng et al., 2014). Given that MDA5 and MAVS are key players in the innate antiviral response, we analyzed their gene expression and identified a significant increase of these genes at day 5 after infection. Thus, while PV does not completely prevent the upregulation of RLRs, it appears to induce a delay in the cellular host response, which is sufficient for the virus to replicate and destroy mature motor neurons.

It is a more general goal of our research team to improve the understanding of viral infections of the CNS by using pluripotent stem cell-derived engineered neural tissues. We have previously investigated CMV infection, and with this study, we report novel data on PV and EV-71 infection, for which the Sabin vaccine strain was used due to biosafety reasons. A comparison among the three highlights important differences (Table 1). The type of neural tissue which is sensitive to a given virus is well-correlated with known clinical features. Poliovirus leads to a paralysis through destruction of motor neurons, a patho-mechanism which is evidently confirmed in our *in vitro* model. HCMV infection occurs mainly in the fetal brain, consistent with the infection of immature neural tissues in our *in vitro* model (Cosset et al., 2015). However, there is a striking phenomenon that requires further investigation: HCMV predominantly infects a small subgroup of early migrating neurons, but leads to substantial changes in gene expression. In contrast, in MN ENT, PV leads to a massive infection and cell death; however, there is a very selective and highly specific impact on expression of a limited number of genes.

**Table 1:** Comparison of the various responses of ENT following viral infection.

		**HCMV**	**Poliovirus**	**Enterovirus-71**
Immature neural tissue		+++	(+)	(+)
Mature neural tissue	Non-directed neural differentiation	n.d.	+	+++
	Motor neuron differentiation	n.d.	+++	+
Changes in gene expression		+++	+	n.d.
Cell death		+	+++	+

*This table presents the difference in terms of virus infection/propagation from elevated +++ to low (+) in the various ENTs. Changes in gene expression and cell death are shown at day 5 and 7 after infection, respectively. n.d., not determined*.

In summary, we have established a pluripotent stem cell-based model of PV infection. The increase in permissiveness upon motor neuron enrichment of engineered neural tissue provides strong validation of our model, as does the induction of cell death. The restricted gene expression profile and delayed innate immune response are notable findings of PV infection, which may provide intriguing clues and opportunities to potential future therapies. Thus, our MN ENT model provides an extremely useful screening tool for therapy development. Despite the availability of a very efficient vaccine, poliomyelitis is yet to be completely eradicated. As noted by World Health Organization: “failure to eradicate polio from these last remaining strongholds could result in as many as 200,000 new cases every year, within 10 years” (https://www.who.int/news-room/fact-sheets/detail/poliomyelitis).

## Data Availability Statement

The datasets presented in this study can be found in online repositories. The names of the repository/repositories and accession number(s) can be found below: Accession number: E-MTAB-9525 Repository: ArrayExpress https://www.ebi.ac.uk/arrayexpress/experiments/E-MTAB-9525/.

## Author Contributions

ÉC designed and supervised the study, designed and carried out experiments, analyzed and interpreted data, and wrote the paper. YH gave conceptual advice provided critical feedback on the study and the manuscript and carried out experiments. SI analyzed the microarray data. P-YD provided critical feedback on the study and the manuscript. CT and K-HK provided critical feedback and gave conceptual advice. All authors proofread the manuscript. All authors contributed to the article and approved the submitted version.

## Conflict of Interest

The authors declare that the research was conducted in the absence of any commercial or financial relationships that could be construed as a potential conflict of interest.

## Supplementary Material

Supplementary Figure 1 **(A)** Schematic representation of air–liquid interface culture principle for MN ENT. The hESCs **(B)** were cultured on Matrigel then aggregated in microwell plates **(C)**. 4-week-old neurospheres **(D)** were plated onto PTFE membrane **(E)** for two additional weeks at the end of which PV-3 infection occurred. **(F)** Immunohistochemistry showed CHAT, HB-9, and ISLET1-immunoreactive cells present in the whole MN ENT (left panel) and lnENT (right panel). Scale bar = 100 μm. **(G)** Schematic representation of air–liquid interface culture principle for neural ENT (lnENT).

Supplementary Figure 2 **(A)** Immunohistochemistry showed enENT infected by EV-71 (left panel) or PV-3 (right panel). Scale bar = 100 μm. Uninfected motor neurons (MN), neural progenitors (NPC), and human pluripotent stem cell (PSC). Immunofluorescence showed PV-3 (green), HB-9, Nestin, and Oct4 (all in red) in MN, NPC, and PSC, respectively. Scale bar = 50μm.

Supplementary Figure 3 **(C)** MN ENT were fixed 5 days after PV-3 infection, sectioned, and double immunostainings were performed with antibodies against PV-3 (green) and CHAT, motor neuron markers (in red). Nuclei were counterstained with DAPI. Scale bar = 50 μm.

Supplementary Figure 4 **(A)** Immunofluorescence showed βIII-Tubulin, and NeuN-immunoreactive cells present in the uninfected MN ENT at day 3 (top panel) and the negative control (mouse and rabbit secondary antibodies; bottom panel). **(B)** MDS plot showing separation of individual samples for uninfected MN ENT (Ctrl MN_5d) and infected MN ENT (PV-3_5d) at day 5 after infection.

Supplementary Table 1 List of primers used for quantitative RT-PCR and RT-PCR.

Supplementary Table 2 List of the differentially expressed gene between PV-3 infected vs. uninfected MN ENT.
