# Supplementary material for: Modeling Poliovirus Infection Using Human Engineered Neural Tissue Enriched With Motor Neuron Derived From Embryonic Stem Cells
Source: Front Cell Dev Biol. 2021 Jan 6;8:593106. doi: 10.3389/fcell.2020.593106 (PMC7815649; doi:10.3389/fcell.2020.593106)
Supplement: Supplementary Figure 1 — (A) Schematic representation of air–liquid interface culture principle for MN ENT. The hESCs (B) were cultured on Matrigel then aggregated in microwell plates (C). 4-week-old neurospheres (D) were plated onto PTFE membrane (E) for two additional weeks at the end of which PV-3 infection occurred. (F) Immunohistochemistry showed CHAT, HB-9, and ISLET1-immunoreactive cells present in the whole MN ENT (left panel) and lnENT (right panel). Scale bar = 100 μm. (G) Schematic representation of air–liquid interface culture principle for neural ENT (lnENT). [file Data_Sheet_1.PDF]

Supplementary Figure 1

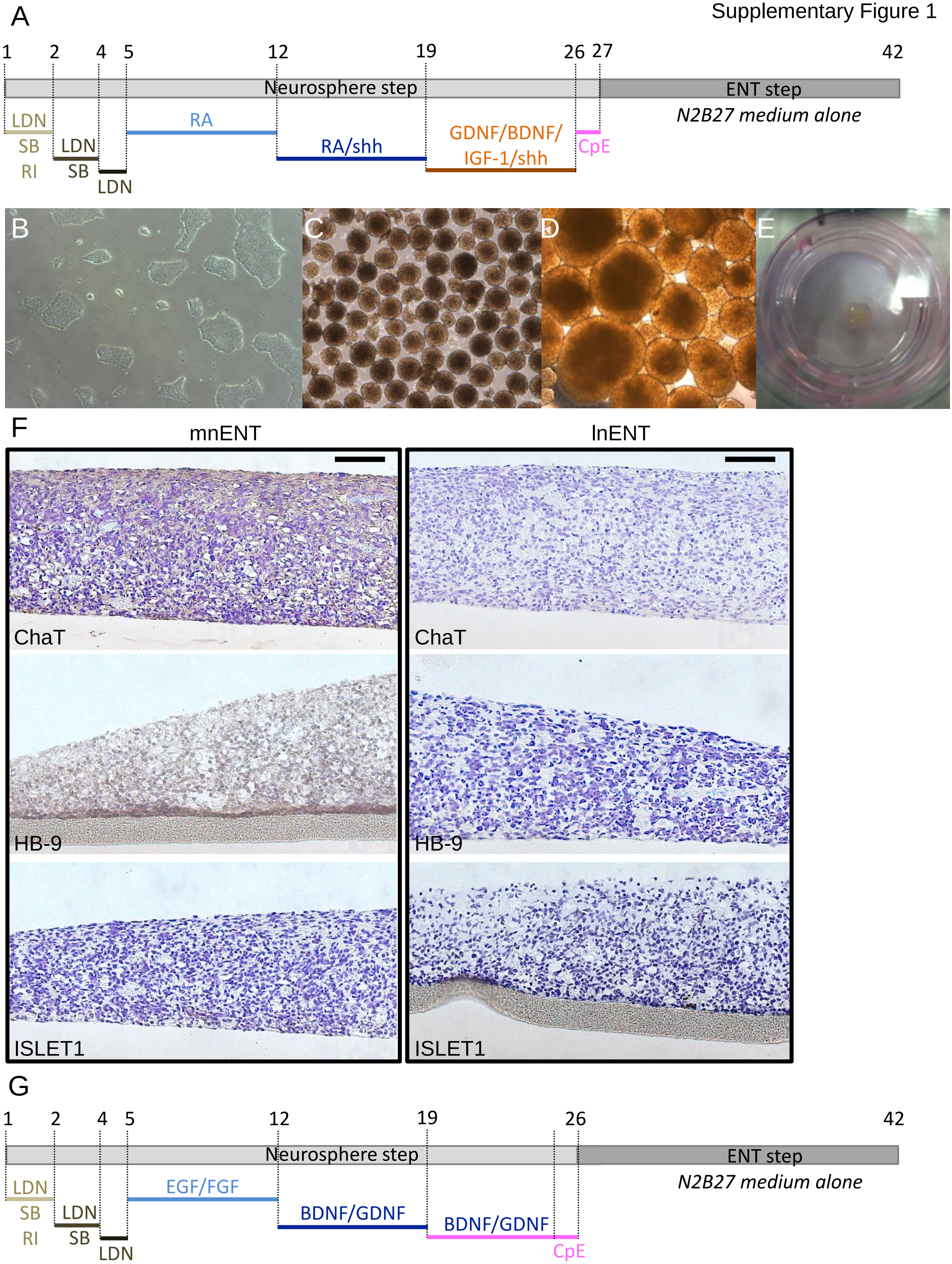

Supplementary Figure 2

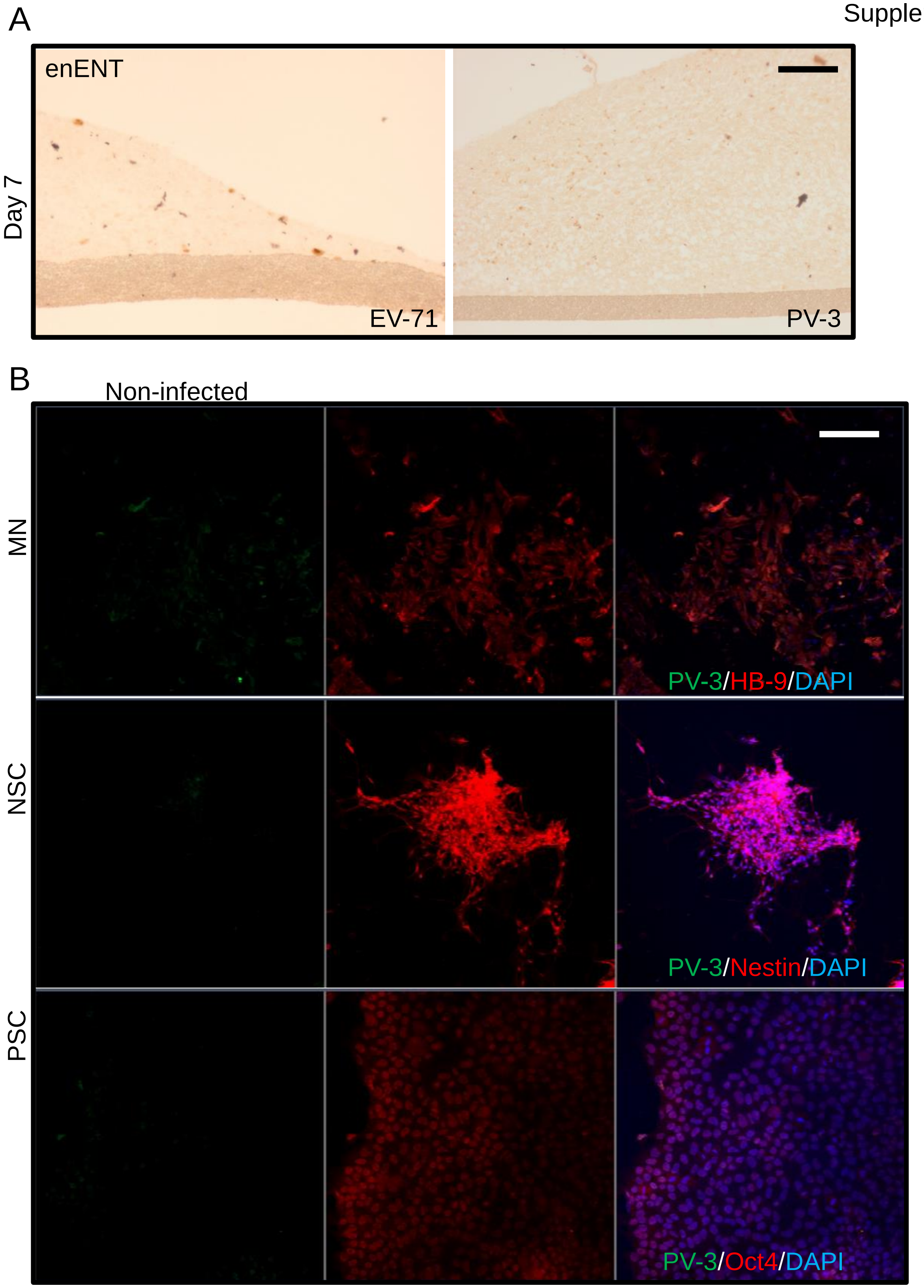

A

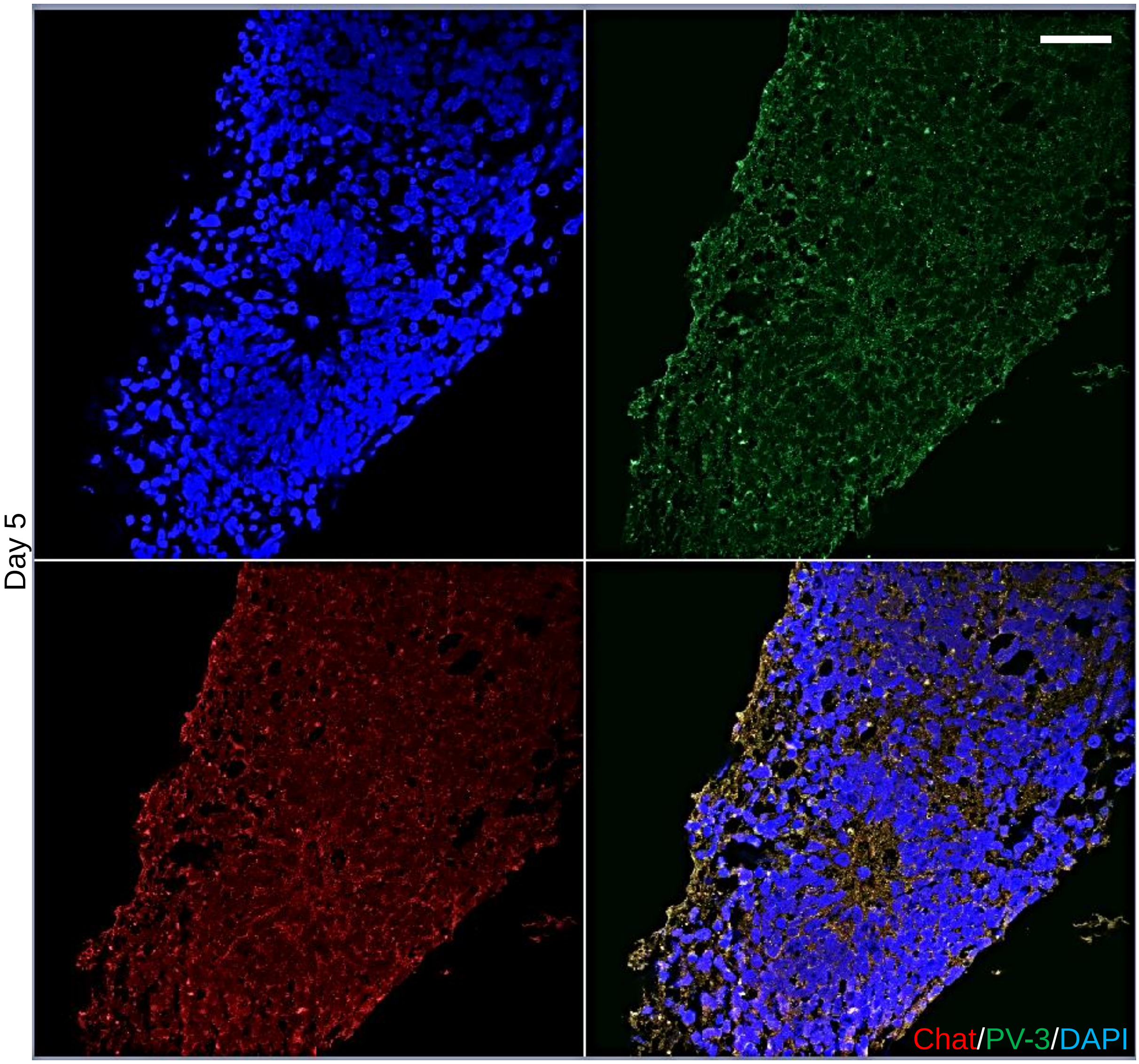

Supplementary Figure 4

A

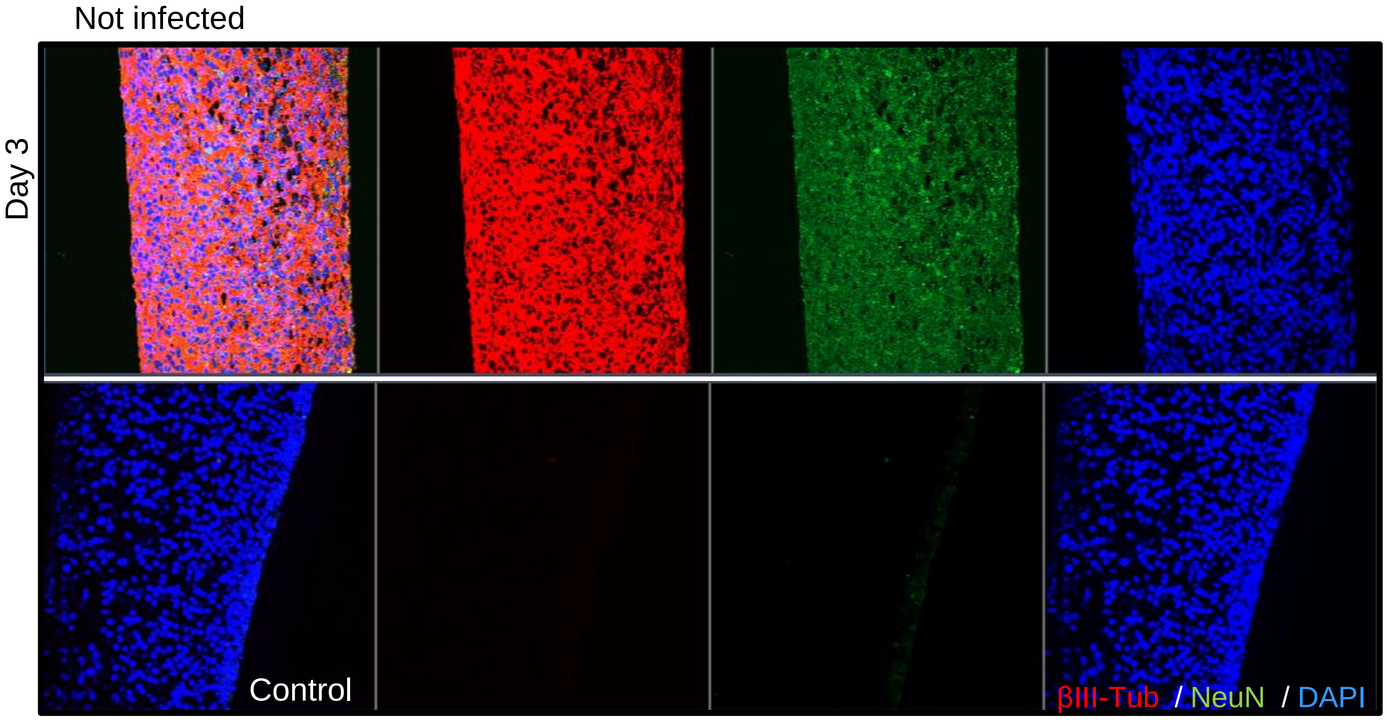

B

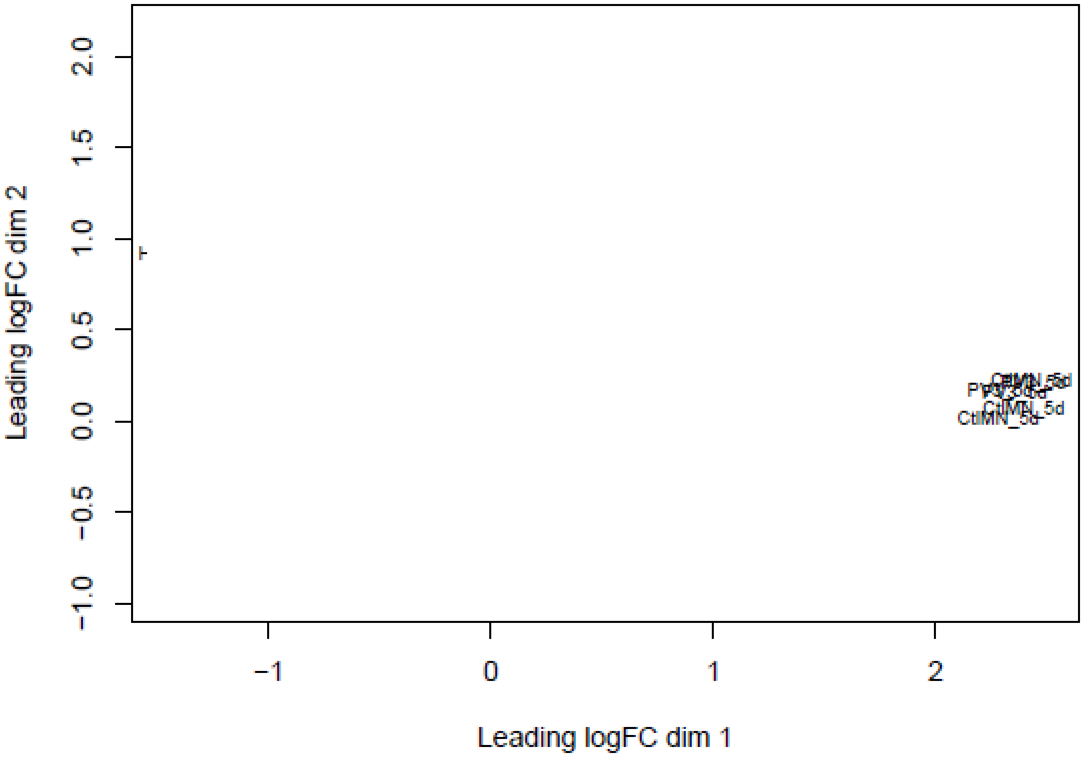

Supplementary Table 1

	Gene		Nucleotide sequence
qPCR	NeuN	Fwd	CGGACAAACGGCTCACTCT
		Rev	GGACCCGCATGAATCGACTAT
	βIII-Tubulin	Fwd	CGGTGGTGGAACCCTACAAC
		Rev	AGGTGGTGACTCCGCTCAT
	Nestin	Fwd	GGAAGAGAACCTGGGAAAGG
		Rev	CTTGGTCCTTCTCCACCGTA
	MDA5	Fwd	AATCATCTTTTGCAGATTCTTCTGT
		Rev	TGTCCAAGACTTTCATCTAAGCA
	MX1	Fwd	GGGAAGGAATGGGAATCAGT
		Rev	CCCACAGCCACTCTGGTTAT
	OAS1	Fwd	CAAGCTCAAGAGCCTCATCC
		Rev	TGGGCTGTGTTGAAATGTGT
	OAS2	Fwd	AGAAGCCAACGTGACATCCT
		Rev	CAAGGGACTTCTGGATCTCG
	ISG15	Fwd	TGCACAAGAGCATCCAGAAC
		Rev	CGGATTCTCTGGGAGATTTG
	ISG20	Fwd	TGCACAAGAGCATCCAGAAC
		Rev	CGGATTCTCTGGGAGATTTG
	RIG1	Fwd	GCTTTGCCTGCTATGAAAGG
		Rev	TTTCCCCTTTTGTCCTTGTG
	IRF9	Fwd	GTACCATCAAAGCGACAGCA
		Rev	AGTTCTGCATGGCATCCTCT
	ALAS1	Fwd	CTCACCACACACCCCAGATG
		Rev	AGTTCCAGCCCCACTTGCT
	EEF1A1	Fwd	AGCAAAAATGACCCACCAATG
		Rev	GGCCTGGATGGTTCAGGATA
PCR	Oct4	Fwd	AGTGCCCGAAACCCACACTG
		Rev	ACCACACTCGGACCACATCCT
	Nanog	Fwd	GATTTGTGGGCCTGAAGAAA
		Rev	TTGGGACTGGTGGAAGAATC
	Nestin	Fwd	GGAAGAGAACCTGGGAAAGG
		Rev	CTTGGTCCTTCTCCACCGTA
	PAX6	Fwd	AACGATAACATACCAAGCGTGT
		Rev	GGTCTGCCCGTTCAACATC
	NGN	Fwd	CTCCATACACTCCAGTGCCAG
		Rev	GCCCACGTAATCCTGATGGT
	Olig2	Fwd	GGACAAGCTAGGAGGCAGTG
		Rev	ATGGCGATGTTGAGGTCGTG
	ChaT	Fwd	CACTGAGCACAGTAGGTCCAC
		Rev	ACACCCAGTTGGCTGTCTTC
	HB9	Fwd	GCTGCAGCGCAAAGAACC
		Rev	CGGGGGCTCGGTATTGTTAT
	MAP2	Fwd	AAAGCTGATGAGGGCAAGAA
		Rev	GGCCCCTGAATAAATTCCAT
	βIII-Tubulin	Fwd	GGCCTGACAATTTCATCTTTGG
		Rev	ACCACATCCAGGACCGAATC
	GFAP	Fwd	AAGAGATCCGCACGCAGTAT
		Rev	AGGTCAAGGACTGCAACTGG
	GAPDH	Fwd	GAAGGTGAAGGTCGGAGTC
		Rev	GAAGATGGTGATGGGATTTC

Supplementary Table 2

Ill_ID	Symbol	Name	logFC	AveExpr	adj.P.Val
ILMN_211	GALR3	galanin receptor 3	4.570944	5.077652	5.9E-10
ILMN_324	RNY4	RNA, Ro-associated Y4	3.448942	8.44669	7.47E-05
ILMN_168	ITM2C	integral membrane protein 2C	-1.8124	11.06319	0.000617
ILMN_176	EGR1	early growth response 1	1.246274	7.978569	0.026036
ILMN_221	DBX2	developing brain homeobox 2	0.644193	4.755297	0.030645
ILMN_177	E2F2	E2F transcription factor 2	1.722131	7.027879	0.035936
